# MRSA model of learning and adaptation: a qualitative study among the general public

**DOI:** 10.1186/1472-6963-12-88

**Published:** 2012-04-02

**Authors:** Rodney E Rohde, Jovita Ross-Gordon

**Affiliations:** 1 Clinical Laboratory Science Program, College of Health Professions, Texas State University-San Marcos, Texas, USA; 2 Department of Counselling, Leadership Adult Education, and School Psychology, College of Education Texas State University-San Marcos, Texas, USA

## Abstract

**Background:**

More people in the US now die from Methicillin Resistant *Staphylococcus aureus *(MRSA) infections than from HIV/AIDS. Often acquired in healthcare facilities or during healthcare procedures, the extremely high incidence of MRSA infections and the dangerously low levels of literacy regarding antibiotic resistance in the general public are on a collision course. Traditional medical approaches to infection control and the conventional attitude healthcare practitioners adopt toward public education are no longer adequate to avoid this collision. This study helps us understand how people acquire and process new information and then adapt behaviours based on learning.

**Methods:**

Using constructivist theory, semi-structured face-to-face and phone interviews were conducted to gather pertinent data. This allowed participants to tell their stories so their experiences could deepen our understanding of this crucial health issue. Interview transcripts were analysed using grounded theory and sensitizing concepts.

**Results:**

Our findings were classified into two main categories, each of which in turn included three subthemes. First, in the category of *Learning*, we identified how individuals used their *Experiences with MRSA*, to answer the questions: *What was learned? *and, *How did learning occur? *The second category, *Adaptation *gave us insights into *Self-reliance, Reliance on others*, and *Reflections on the MRSA journey*.

**Conclusions:**

This study underscores the critical importance of educational programs for patients, and improved continuing education for healthcare providers. Five specific results of this study can reduce the vacuum that currently exists between the knowledge and information available to healthcare professionals, and how that information is conveyed to the public. These points include: 1) a common model of MRSA learning and adaptation; 2) the self-directed nature of adult learning; 3) the focus on general MRSA information, care and prevention, and antibiotic resistance; 4) the interconnected nature of adaptation; and, 5) the need for a consistent step by step plan to deal with MRSA provided at the time of diagnosis.

## Background

Methicillin-resistant *Staphylococcus *aureus (MRSA) first emerged as a serious infectious threat in the late 1960s as the bacterium developed resistance to the synthetic form of penicillin known as methicillin [1]. Although the *Staphylococci *bacteria, including MRSA, commonly colonize the skin of healthy people, often posing little to no threat, these bugs are quick to exploit any opportunity to invade wounds, nasal passageways, or mucosal membranes where they can rapidly produce infections that can become life threatening. It is not surprising then, that MRSA has been the focus of intense scientific and political interest around the world [2,3] and has frequently been labeled as a *superbug *in the popular media [4].

As the number of MRSA infections acquired both within healthcare facilities and, more recently, in community settings that bring large numbers of people into close proximity have increased, research has begun to focus on levels of public awareness and misperceptions connected with MRSA. In particular, this decade has produced a number of significant studies in the United Kingdom and Europe that have investigated public perceptions and the role played by the popular media as purveyors of information. As recently as 2006, a study conducted in the UK [5] found that 68% of the lay people they surveyed acquired their knowledge of MRSA from a combination of television and newspapers. An earlier qualitative study reported a lingering level of confusion in patients being treated for a MRSA infection, that persisted even after information about the infection had been provided by healthcare professionals [6]. These findings are further supported by a 2007 investigation into public awareness and attitudes which reported "the media [continues to act] as a conduit between medical and lay knowledge and . . . is the main source of public information about resistant infections."[7]. Finally, in perhaps one of the largest empirical studies of MRSA to date, conducted in the UK, researchers found that misperceptions surrounding MRSA tenaciously persist. This 2009 survey of 1,000 respondents found higher levels of public awareness than anticipated, and noted the primary source of information continues to be the media. But, perhaps most notable, the researchers report that "no one in [their] sample mentioned the contributions to MRSA of antibiotic prescribing by doctors or patient use of antibiotics" [4].

This study hopes to build on the findings of the research discussed above by delving deeper into the learning experiences of people who have lived through a MRSA infection in order to improve the practical management and outcomes of this disease.

## Methods

### Paradigm

The aim of developing a deeper and more comprehensive understanding of *how *and *why *people learn [8] about MRSA was the impetus for this study. The researchers selected a qualitative approach in order to focus on *individual lived experience *[9]. The unique strengths of this kind of exploratory research include: 1) "the value of context"; 2) "the [search] for a deeper understanding of the phenomenon under study"; and 3) the belief that "people [use] meaning [to] define their situations" [9]. In other words, because the study was situated in a social science genre, the research methodology best suited to the analysis was also situated in the social sciences.

The principles of constructivist theory governed the design. Constructivist theory holds that individuals develop deep, subjective meanings based on their experiences. Typically, these meanings are negotiated socially through a process of discussion, reflection, and interaction. These meanings then often become part of the cultural and social norms that govern values and behaviours [10]. In order to explore the complexities associated with these subjective meanings, constructivist theory uses broad, general, open-ended questions to encourage participants to provide rich descriptions of their experiences. This provides the opportunity for participants to paint a picture of a world unknown to the reader [11-13]. Participants' descriptions of their experiences with MRSA allow the reader to understand how they constructed knowledge about a new phenomenon, the learning strategies they used, and how they adapted to a MRSA diagnosis.

The researcher's objective then becomes one of interpretation, or of making sense of the information provided by the participants. This process is referred to as inductive analysis as the researcher distils patterns of meaning that in turn construct theory [10].

Sample-size in qualitative research depends on a number of factors. However, recent qualitative studies tend to range from four to ten participants [9]. The first objective is to ensure a reasonable degree "of variation in the phenomenon, settings, or people" [14]. Secondly, the sample size needs to be large enough to achieve saturation, or the point at which no new information is being provided by additional participants [15]. Qualitative researchers have validated data saturation occurring with as few as six participants, and typically within the first 12 interviews.

### Participants

These criteria governed the purposive selection of participants and ensured that each individual: 1) was a member of the general public who had contracted MRSA in a non-healthcare related setting; 2) had a positive MRSA diagnosis within the last year, but at least one month prior to the interview; 3) was 18 years or older; 4) was not hospitalized; 5) was not in a long-term care facility; and, 6) was not employed in direct patient care.

Initial referrals for this study came from a university health center as a random "first come, first served" basis. Recruitment fliers detailing the study were placed in plain view in the health center. These individuals, in turn, provided subsequent leads for other potential participants. In the end, ten individuals were selected for inclusion. The three men who participated in the study ranged in age from 21 to 73. All of them were Caucasian; one had completed an M.B.A., another had a B.S., and the third had some college education; at the time of the research, one was unemployed and two were retired. The seven women who participated ranged in age from 21 to 65. One of them was Vietnamese, the rest were Caucasian. All of them had some college, three had completed bachelor's degrees, one had a master's degree, and one had an associate LVN. During their participation, two of the women were retired, one was working full-time, three worked part-time, two were retired, and one was unemployed. Names used throughout this document are pseudonyms selected by the participants. To the degree possible, an effort was made to select a sample diverse in ethnicity, gender, and age in order to produce the maximum variation among participants so as to increase the robustness of the conclusions and enhance the likelihood of transferability to other groups or individuals. Nonetheless, the researchers recognize the inherent limitations relative to generalization to a wider population due to the small sample size.

### Data collection

Data was collected using private, confidential, face-to-face initial interviews. In one instance, because of distance, the initial interview was conducted by telephone. Subsequently, telephone interviews were used when clarification or follow-up information was needed. A set of pre-determined questions guided these discussions. Open-ended questions were used to create a semi-structured, nondirective environment that encouraged participants to use their own words to describe their experiences, and to explore details that held particular meaning for them. These conditions reflect the nature of constructivist research which holds the premise that individuals are experts on their own experiences, and that evidence is derived from first-person reports of life experiences [8,11,13,15].

### Data analysis

The tape-recorded interviews were transcribed verbatim and pseudonyms were assigned for confidentiality. Transcripts were then coded for commonalities, and a cross-reference matrix was developed to screen for these commonalities. Secondly, transcriptions were read and reread from a more global perspective to identify and index categories and themes as they developed around particular phrases, incidents, or types of behaviours [15].

Analysis began with the first interview. It was inductive, and ongoing throughout the interview phase. Concurrent analysis allows the qualitative researcher to identify the data saturation point [15]. In this study, saturation occurred at the eighth interview. Two additional interviews were conducted to ensure no additional themes emerged.

As the analysis progressed, the principles of qualitative research: 1) credibility; 2) transferability; 3) dependability; 4) confirmability, and; 5) triangulation, were constantly scrutinized. This inquiry was conducted in such a manner as to address each of these elements.

### Ethical concerns

The research proposed in this qualitative study was approved by the Texas State University-San Marcos Institutional Review Board (2009z4233). In accordance with IRB requirements, participants were informed of their rights and asked to read and sign an informed consent form. They were advised they could stop the interview or drop out of the project at any time. None chose to do either. No participant indicated that they felt uncomfortable discussing their experiences with MRSA infections, and all expressed willingness to tell their stories. All recordings, computer files, transcripts, and paperwork were identified only by the participant's pseudonym and were kept in a locked file cabinet only accessible by the primary researcher.

## Results

During the interviews the participants reflected on their experiences with MRSA and how it impacted their lives. Their willingness to share their stories offers us the opportunity to learn about their walk with MRSA, their fears and triumphs, and their insights about the world of emerging antibiotic resistant infections. Their stories present a combination of pain, *aha moments*, and an inner search for strategies to help them learn about and adapt to this growing public health problem. All were happy to share what they had learned in their personal journey.

With respect to age, the group divided into young adults under the age of 25 (four of the participants), and older adults over the age of 50 (the remaining six participants). Although there were commonalities between the two groups, the younger group exhibited a degree of apathy and passive learning that was different from the older group. The older group also demonstrated more signs of *reflective hindsight *and an *appreciation for life *due to their encounter with MRSA.

Most of the individuals in this study acquired their knowledge and learning about MRSA after their diagnosis. In general, all of the participants were aware of MRSA and/or staph but they lacked detailed information about the organism and its dangers. They acquired information and knowledge through a variety of sources, although printed media and the internet served as the two primary vehicles for accessing information. Learning was primarily self-directed, experiential, and in some cases, transformational.

### Learning - *"I guess everything changes when it happens to you*"

Every participant found learning was a critical step in their production of knowledge about MRSA, and that knowledge affected their adaptation to the infection. Within the broad theme of learning, three subthemes emerged. These included: a) Experiences with MRSA; b) What was learned; and, c) How learning occurred. Following is a brief discussion of each of these subthemes.

### Experiences with MRSA

The participants experienced several common threads in their MRSA journeys. All talked about the physical pain, ranging from bad to severe, both prior to and after the diagnosis. *The worst pain I have ever felt in my entire life *[Becky] *. . . on a scale of 1 - 10, probably a 9 *[Erin] *. . . it put me in a really bad mood because it was so much pain, constantly *[Erin].

Another common thread was the emotional factors associated with MRSA. The participants discussed a range of emotions including trust, anger, anxiety, worry, fear, depression, frustration, and embarrassment associated with feelings of being stigmatized. *I was scared to death *[Edward] *. . . I was so mad and upset *[Aaron] *. . . I just paid $500 for this visit and you didn't tell me anything I don't already know *[Trene] *. . . other people were just really scared to come around me *[Dora] *. . . even [my] family wouldn't give [me] hugs *[Dora] *. . . the stigma of staph is just like, oh, you just roll around in dirt all day long and you don't clean yourself, and you have all these nasty, open sores *[Trene] *. . . it's [MRSA] always in the back of my mind *[Mary].

Interactions with healthcare personnel, whether positive or negative, were an important experience for every participant. The interaction was on multiple levels with a variety of healthcare providers - primary care physicians, ER [emergency room] doctors, specialists (infectious disease, orthopaedic, etc.), nurses (both in the hospital and at home), physical therapists, office staff, and internists. *I thought that I was getting the best treatment and I knew they were doing the best they could *[Irene] *. . . you get some confidence when everybody says treat it the same way [consistent message] *[Irene] *. . . we never saw the doctor - the nurse [told us] the diagnosis and then walked away *[Edward] *. . . they didn't even think about staph, even after I [told] them I went to the gym, and had gotten tattoos - it made me question their knowledge *[Aaron].

### What was learned?

The content of learning included three main areas: 1) general MRSA information; 2) MRSA care and prevention; and, 3) antibiotic resistance.

In general, all of the participants were aware of MRSA but lacked detailed knowledge about the microbe. For instance, all of the participants knew what a 'staph' infection was but they learned MRSA was a specific type of staph infection that was resistant to treatment. MRSA colonization and reservoirs were also major content areas of learning for everyone. *It can live, you know, other places than in your body, on the walls, in carpets, and they said I could carry it in my body *[Dora] *. . . people are kind of familiar with infections, but it's [MRSA] in a different league by itself *[Alvin] *. . . [they asked] all these questions about my lifestyle, what kind of shorts or pants I work out in, do I wait to shower after I've been sweating, do I shower too much *[Becky] *. . . I though it [MRSA] was just some big, bad germ that lurked in the dark hollows of the hospital, but it's just a germ out there [in the community] *[Edward].

The second major component of learning was about the care and prevention of MRSA. Once the MRSA diagnosis had been made, a consistent message about wound care, medication, and how to prevent the spread of MRSA was delivered by the respective healthcare professionals involved in each case. The participants seemed to be startled and surprised by the ubiquity of MRSA, especially with respect to its presence in the environment outside of healthcare. *It's [MRSA] always present *[Alvin] *. . . you sit away from people, you try not to touch stuff, you wash your hands so much they're wrinkled *[Alvin] *. . . we cut down on our traveling *[Alvin] *. . . my awareness is totally heightened *[Alvin] *. . . we bought a sack of white washcloths - those were mine - when I [finished using one] we'd put it in bleach water - nobody else used that washcloth *[Edward] *. . . we would wash it, clean it, change the dressing, and put everything in a bag, tie it in a knot, and put it in another bag [before] we threw it away *[Edward] *. . . I learned to not be around people as much in a close, close quarter and to wash your hands a lot more and to definitely take a bath every day *[Erin].

The ability of microbes to undergo mutations which leads to resistant strains of the organism has been well-documented in the world of microbiology. But, in the general community, this topic is commonly misunderstood. Most of the participants learned about the specific types of antibiotics used for MRSA infections and, in general, why resistance had occurred for this organism. Each of the participants also discussed learning about the importance of having a culture done, and a subsequent antibiotic susceptibility test to treat the infection appropriately with the correct antibiotic. *I realized having a firm culture diagnosis is essential *[Mary] *. . . I believe it stems a lot from a misuse of antibiotics - overuse of antibiotics - plus a certain germ can mutate into something else *[Nell] *. . . where it [antibiotic] doesn't do its job because you've had so much of it sometimes - so, your germ acclimates to what you've been putting in your body - the germ is going to survive somehow *[Edward].

### How did learning occur?

Learning by the participants in this study was achieved primarily through two channels - people and media. People, both social networks and healthcare professionals, were important sources of information about MRSA. Among this group, a variety of family members played crucial roles in helping all of the participants learn about MRSA. *[My] mom did a whole bunch of research on it [MRSA] - I honestly don't know what I would have done if my mom wasn't there *[Aaron] *. . . [if we had known what we know now] I know my wife would have been very cognizant of my wound and probably would have insisted on a culture *[Edward] *. . . she [wife] gathered information *[Edward] *. . . my wife played a significant and crucial role in [my] learning *[Edward]. Friends and peers were another important layer in the participants' learning from their social network. *My friends told me it was staph because they looked at it and said it was probably staph *[Erin] *. . . if someone mentions MRSA, or I know a friend or relative who has it, I'll ask some questions just to see what their course of treatment is *[Nell].

Healthcare professionals also played a very important role in the learning process. Often, these professionals laid the groundwork for how the participants pursued their overall learning about the disease and, they seemed to influence the participant's attitude and belief in what they learned about the infection. *They [doctors] were good - we'd ask a question and they'd tell us what they knew - the more information we got together, the calmer we became *[Alvin] *. . . they [home health nurses] made it very, very emphatic to me that I had to move *[Irene] *. . . the state nurse came out and educated me *[Dora] *. . . the doctor did an outstanding job - he discussed the importance of why he was culturing the bacteria and then the critical need to perform an antibiotic susceptibility test *[Becky] *. . . he [doctor] made me very comfortable - I asked him a million questions *[Becky]. Interestingly, each of the participants cited both positive and negative interactions with the different healthcare professionals. So, while learning did occur via the various healthcare professionals, in some instances an opportunity was missed because of the environment that was created from a negative encounter.

Printed media, primarily in the form of hand-outs or brochures, provided an important source of information about MRSA Even signage in hospitals and other medical facilities played a role in learning about the precautions to take, and hygiene with respect to an infection. In addition to printed material, six of the participants took advantage of electronic sources to learn more about their condition and the microbe. Some of the websites they used included the Centers for Disease Control, the Mayo Clinic, WebMD, Dr. Oz, and even blogs by others who had experienced a MRSA infection. Some of the participants pointed out they used television and radio to alert them to interesting and informative information about MRSA that they would then look-up on the internet for additional detail.

### Adaptation - "*People make the difference*"

This investigation asked each of the individuals to reflect on their journey *living with MRSA*. Their frank answers to the interview questions provided the following insights into how they adapted to their situations.

### Self-reliance

As with any illness or disease, a diagnosis of MRSA is often associated with an adaptation to the disease or condition over time. As learning occurs, the individual often exhibits strategies and mechanisms to live with the challenges that come with having a health concern. Most of the participants felt that being proactive with how they handled the impact of MRSA was an important factor in adapting to the condition. *Don't be afraid to ask questions, ask what they're doing, why they're doing it, because that way you get educated in what to do also *[Edward] *. . . if you've got any kind of problem, any kind of problem, you ask that doctor to take a culture and stay on top of it *[Edward] *. . . life is precious and we need to do everything we can right now *[Nell] *. . . it led me to wonder about people's responsibility and mindset about, you know, protecting society, because we are totally dependent on that *[Mary] *. . . any bites we get, ants, mosquitos, anything, we go and put alcohol on them immediately *[Dora] *. . . keep your fingernails clean, under your fingernails, wash your hands, wash your hands, wash your hands *[Dora] *. . . when these flare up we basically quarantine ourselves quite a bit *[Alvin] *. . . it put me a little over the top with cleanliness *[Trene] *. . . my husband and I don't share towels anymore, we don't share razors, we don't even share toothpaste, it has changed my life in those regards *[Mary]. These comments show that the participants take MRSA seriously, they take responsibility for their own wound care as well as limiting their activities to minimize exposure for others, and they have modified their attitudes towards hygiene, infection control, and their environments.

### Reliance on others

The relationships that participants formed with family, friends, and healthcare professionals were a crucial component to the adaptation process. The message of others helping them through the MRSA experience was echoed by all participants many times in this study. *My wife goes with me on almost every visit - you need that extra set of ears there and somebody taking notes *[Alvin] *. . . [my] wife helped [me] through depression during the long antibiotic treatments *[Edward] *. . . I've been around doctors my whole life and my parents have just valued doctors and their opinions *[Trene] *. . . I have four good friends that are physicians, and a cousin who I am very close to that is a physician - it's a definite plus, a definite plus *[Irene] *. . . it's easy, you know, to have these people around you that you are close to that if you have a problem that feel like you can discuss it with, whether it be [daughter], whether it be a friend, whether it be a family member *[Irene].

The participants' reliance and trust in healthcare professions was also evident in several of the interviews. These individuals who created a comfort zone for their patients, who were patient with their questions, and who provided thorough and comprehensive information on the course of treatment were an invaluable resource for the participants. Honest, unhurried doctor-patient interactions were an important mechanism that created trust and helped individuals adapt to a MRSA infection.

### Reflections on the MRSA journey

As might be expected, those who had a more severe MRSA infection or had multiple reocurrences with the infection were often more insightful about their journey. Reflection occurred as part of the interviews primarily when the participants were questioned about (a) living with MRSA and how it may have changed their life, (b) advice they would give to someone diagnosed with MRSA, and (c) advice they would give to healthcare professionals to help individuals diagnosed with MRSA. These reflections were often cited by the participants as being helpful for their understanding and adaptation to their MRSA experience.

Living with MRSA seemed to influence the participants to become advocates for educating others about their experience. By educating others about this growing public health threat, the participants felt like it helped them to warn others while also letting them work through the adaptive phase of their infection. Each of the ten participants was in some way, large or small, an advocate for educating others about the dangers of MRSA. By being active in telling their story, the participants found it therapeutic for their own healing. *Make sure you know what you're dealing with, don't take it lightly *[Alvin] *. . . be aware of [your] environment - clean everything for sure *[Trene] *. . . don't be passive with your physician *[Trene] *. . . do the culture *[Alvin] *. . . find out first what strain of MRSA it is *[Edward] *. . . there is a protocol to make sure that the loved ones are taken care of and protected from it so that you don't spread anything *[Edward] *. . . if you've got any kind of problem, any kind of problem, you ask that doctor to take a culture and stay on top of it *[Edward] *. . . don't skip on the pills [antibiotics], even if you think your infection's getting better *[Erin].

Most of the participants discussed how healthcare professionals could improve the interaction between the two parties by offering examples of what they felt had worked or not worked for them during their experiences. By doing so, the participants all reiterated that the reflective advice was helpful to them *that maybe our story will help others*.

Several of the participants also discussed how spirituality and prayer helped them get through their respective MRSA experiences. For these participants, their relationship with God and their usage of prayer helped them to adapt and accept the trials they were going through. It also gave them strength to endure the pain and emotional rollercoaster they were on during the infection process.

## Discussion

**Figure 1:**
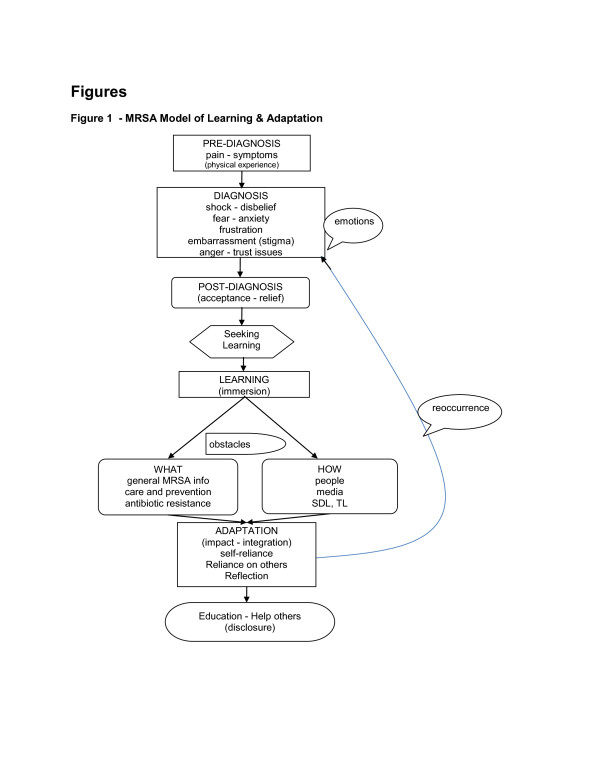
**MRSA Model of Learning & Adaptation**.

The experiences of the ten participants in this study combined to create a common model of how a person with MRSA in the general public learns about and adapts to the infection. In their experiences, learning (see Figure 1) is initiated with a pre-diagnosis, and then continues in order, through diagnosis, post-diagnosis, learning, adaptation, and disclosure/closure. Based on the findings in this study, initial experiences with MRSA typically follow these steps, in sequence, although a reocurrence may move the individual back upstream to an earlier stage in the learning and adaptation sequence, indicating that learning tends to be a fluid process. We believe this is the first documented model to explain the MRSA experience. It is clear both from this study and from the associated literature, that the nature of adult learning during a MRSA experience is primarily self-directed, and for some, transformational. Further, learning occurred via two channels - people (both social networks and healthcare professionals) and media (print and electronic). Lastly, an association with social representation theory [1,16] was found relative to how participants with MRSA were influenced by the media in regards to anchors and objectification of the condition.

Through their experiences, the participants developed a wide range of knowledge about MRSA. Key learning content was associated with the difference between regular staph and MRSA, preventive measures to minimize exposure, wound care, avoidance of transmission to others, and the development of antibiotic resistance. The content of each of these learning areas was distinct and contextual for each individual participant. For example, some participants had a single MRSA infection while others had multiple reocurrences. Also, some participants were in good general health while others were severely immunocompromised. Obviously, these factors led to different experiences with learning content due to the individual severity of infection. The participants often had poor knowledge about antibiotic resistance prior to their MRSA diagnosis, which is consistent with the literature associated with both healthcare-acquired MRSA and community-acquired MRSA [17].

Perhaps most importantly, almost all participants had important advice for healthcare professionals about the need for a *consistent message *and a *MRSA plan*. The embedded features of this plan were that people make the difference in these life-changing diagnoses. Likewise, all participants echoed the need for consistency of MRSA information and a step-by-step plan to manage the condition.

## Conclusions

The implications for practice and research based on the literature and results of this study indicate a need to address issues of how the general public discovers, learns about, and adapts to antibiotic resistant infections, especially MRSA. Likewise, this study emphasizes the critical importance of informing healthcare professionals and health educators (e.g., universities, schools, and other related institutions) about the need for better programs of patient education and continuing education surrounding the pre and post diagnosis of MRSA infections.

### Practice

Current patient education programs about antibiotic resistant infections need to be revised. Particular attention should be paid to the following areas: a) the patient-healthcare provider interaction should intentionally cultivate an open and non-threatening environment so that learning can occur; b) the delivery of critical information about the importance of having a MRSA diagnosis based on *laboratory culture and antibiotic susceptibility testing*; c) specific education on *what a MRSA infection looks like*, including images/pictures and *MRSA stories *for patients; d) the use of podcasts, digital video, and other electronic media to provide patient education beyond the initial MRSA diagnosis; e) specific education about infection care, control, and prevention to themselves and others; and, f) guidance for individuals about sources of information and the credibility of sources.

### Research and theory

Study findings indicate the need for further research on this subject. Antibiotic resistant infections will continue to increase, both in numbers and types, if current predictions hold true. Further research on this subject will help in better defining and understanding how the lay public accesses, interprets, learns, and adapts to MRSA infections and will aid healthcare professionals and health educators in planning for continuing education and public health education programs. Additionally, it is important to continue to document and capture the stories and lessons learned from the general public. These stories tend to be under-reported in comparison to accounts from the healthcare environment and add important, often overlooked aspect about MRSA infections and their impact on society.

The results of this study add to the overall body of knowledge concerning how adults learn and adapt to a MRSA infection. Importantly, it is the first study to document a model for adult learning and adaptation to this growing healthcare threat in the general public.

### Final thoughts

We live in an era of rapidly growing, emerging, and re-emerging infectious disease both in the healthcare environment and in the general community. Based on the literature and this study, explanations for prevention and control of outbreaks in the general public and why other public health campaigns may have failed appears to be linked to *what *and *how *people learn about MRSA. The implications for healthcare providers and health educators to implement new and effective strategies for people to learn about their health conditions may be more pertinent than ever. The findings of this study should be used to develop specific health education and promotion activities for those at greater risk for acquiring MRSA or who are currently colonized. It can identify antibiotic resistance mis-information and poor educational trends, assist healthcare officials in the control and prevention of MRSA, and increase our understanding of the educational needs of individuals who acquire MRSA infections and their subsequent actions to cope with their condition. This research contributes to the fields of health education, public health, health social science, infectious disease, and epidemiology. By understanding individual perspectives on MRSA, we can try to better translate personal health knowledge construction to public health personnel and policymakers. Thus, the findings in this study should be used to build better planned and more successful public health campaigns against antibiotic resistance in general, and MRSA in particular.

## Competing interests

RER is an Associate Editor for BMC. This relationship has no bearing on the research presented herein. The authors declare that they have no competing interests.

This study was supported financially by a grant from the American Society of Clinical Laboratory Science (July, 2009); participants were compensated at a rate of $50 for the first interview, and an additional $50 for any or all follow-up interviews.

## Authors' contributions

RER and JRG designed the study. RER conducted the interviews, transcribed the tapes, and made the preliminary analysis. Both authors contributed to the final analysis of the interviews. Both authors have read and approved the final manuscript.

## Pre-publication history

The pre-publication history for this paper can be accessed here:

http://www.biomedcentral.com/1472-6963/12/88/prepub

## Supplementary Material

Additional file 1 **Appendix A**. Recruitment and Scheduling Flier. Appendix B. Letter of Introduction/Consent Form. Appendix C Interview Guide. Appendix D - Cross Reference Matrix. Click here for file

## References

[B1] Washer P Joffe H The "hospital superbug": Social representations of mrsa Soc Sci Med 2006 63 2141 2152 10.1016/j.socscimed.2006.05.018 16782254

[B2] Invasive mrsa http://www.cdc.gov/ncidod/dhqp/ar_mrsa_Invasive_FS.html

[B3] Darzi AZ Our nhs our future: Nhs next stage review interim report 2007 London: Department of Health

[B4] Easton PM Marwick CA Williams FL Stringer K McCowan C Davey P Nathwani D A survey on public knowledge and perceptions of methicillin-resistant *staphylococcus aureus* J Antimicrob Chemother 2009 63 209 214 18984646 10.1093/jac/dkn447

[B5] Gill J Kumar R Todd J Wiskin C Methicillin-resistant *staphylococcus aureus: Awareness and perceptions* J Hosp Infect 2006 62 333 337 10.1016/j.jhin.2005.09.009 16377028

[B6] Newton JT Constable D Senior V Patient's perceptions of methicillin-resistant staphylococcus aureus and source isolation: A qualitative analysis of source-isolated patients J Hosp Infect 2001 48 275 280 10.1053/jhin.2001.1019 11461128

[B7] Hawkings NJ Wood F Butler CC Public attitudes towards bacterial resistance: A qualitative study J Antimicrob Chemother 2007 59 1155 1160 10.1093/jac/dkm103 17449888

[B8] Creswell JW Qualitative inquiry and research design: Choosing among five traditions 1998 Thousand Oaks, CA: Sage Publications, Inc.

[B9] Marshall C Rossman GB Designing qualitative research 2006 4 London: Sage Publications

[B10] Creswell JW Research design: Qualitative, quantitative, and mixed methods approaches 2009 3 NE: University of Nebraska-Lincoln

[B11] Denzin NK Lincoln YS Denzin NK, Lincoln YS Introduction: The discipline and practice of qualitative research Handbook of qualitative research 2005 3 Thousand Oaks, CA: Sage 1 32

[B12] Maxwell J Qualitative research design: An interactive approach 2004 2 Thousand Oaks, CA: Sage

[B13] Strauss AL Corbin J Basics of qualitative research: Techniques and procedures for developing grounded theory 1998 2 Thousand Oaks, CA: Sage

[B14] Dobbert ML Ethnographic research: Theory and application for modern schools and societies 1982 New York: Praeger

[B15] Patton MQ Qualitative research and evaluation methods 2002 3 Thousand Oaks, CA: Sage

[B16] Moscovici S Farr RM, Moscovici S The phenomenon of social representations Social representations 1984 Cambridge: Cambridge University Press 3 70

[B17] Gould DJ Drey NS Millar M Wilks M Chamney M Patients and the public: Knowledge, sources of information and perceptions about healthcare-associated infection J Hosp Infect 2009 72 1 8 10.1016/j.jhin.2009.01.024 19282049

