# Supplementary material for: MRSA model of learning and adaptation: a qualitative study among the general public
Source: BMC Health Serv Res. 2012 Apr 2;12:88. doi: 10.1186/1472-6963-12-88 (PMC3342089; doi:10.1186/1472-6963-12-88)
Supplement: Additional file 1 — Appendix A. Recruitment and Scheduling Flier. Appendix B. Letter of Introduction/Consent Form. Appendix C Interview Guide. Appendix D - Cross Reference Matrix. [file 1472-6963-12-88-S1.DOC]

**Appendix A – Recruitment and Scheduling Flier**

***MRSA Knowledge, Learning, and Adaptation Study***

Be part of an important public health study.

- Are you over the age of 18?
- Have you been diagnosed with Methicillin Resistant *Staphylococcus aureus* (MRSA) within the past year?

If you answered YES to these questions, you may be eligible to participate in an important research project.

The purpose of this study is to investigate how people in the community who have MRSA develop their understanding and knowledge about their condition. The intention is to explore how members of the general public who have a “need to know” discover, learn, and adapt to MRSA.

Participants will receive an incentive payment. No medication will be given.

Participants will be interviewed about their MRSA experience for 1-2 hours. A potential follow-up interview may be required.

Adults over the age of 18 with a diagnosis of MRSA at least one month prior to the interview and within the past year are eligible.

This study is being conducted at Texas State University, 601 University Drive, San Marcos, TX 78666 USA

Please call Rodney Rohde at (512) 245-2562 or email at [rrohde@txstate.edu](mailto:rrohde@txstate.edu) for more information.

*This study has been approved by the Texas State University-San Marcos Institutional Review Board (IRB* 2009z4233)

**APPENDIX B – LETTER OF INTRODUCTION / CONSENT FORM**

IRB# 2009z4233

**Purpose**

The purpose of this research study is to find out how people learn and adapt after they have been diagnosed with a resistant form of a bacterium called *Staphylococcus aureus*, often referred to as MRSA or “mersa”. My name is Rodney Rohde and I am a Ph.D. student in the Education Department (Adult Professional Community Education) at Texas State University – San Marcos. I am also an Associate Professor at Texas State in the Clinical Laboratory Science Program. You are being asked to be in this study because you are from the general community, an adult above the age of 18, and have been told you have MRSA. I hope to have 10-12 people in this study. If you have any questions, please ask me. You can contact me (512-245-2562, rrohde@txstate.edu) or my advisor, Dr. Jovita Ross-Gordon (512-245-8084, jross-gordon@txstate.edu).

**Voluntary Participation**

Participation in this research project is completely voluntary. **You** are deciding if you want to be a part of this project. You should be over the age of 18 and not involved in the healthcare industry. You will be asked to participate in an interview which will be audio recorded. This will take approximately 60 to 90 minutes of your time. You may also receive a follow-up phone call or email at a later date to clarify your answers from the interview or possibly to ask a few additional questions. You can choose not to participate in this study. Just tell me that you do not want to participate. If you decide to participate now and later decide you don’t want to remain in the study, that’s okay. In that case, I will not use your interview information. If at any time during the study, you feel uncomfortable you may quit. Participation is entirely voluntary and you may withdraw from the study at any time without prejudice or jeopardy to your standing with this university or any other organization.

**Confidentiality**

I will be the only one who knows you were in this study. I will not share your name with anyone. Once all of the interviews are completed and the data is analyzed, my dissertation will be based on the study and further educational conference papers or professional journal articles may be generated. No indications of your actual identity will be made in any of these documents. Pseudonyms will be used for the participants in an effort to preserve your confidentiality. I will change any identifying information or revealing details. However, if you are interested in the findings of this research study, a summary of the findings will be provided to you if requested. You may choose to offer your email as a means of obtaining the completed results. Your email address will not be used to link you to the results. The audio tapes will be erased within 12 months. Should you decide to, you may withdraw from the study at any time.

**Potential Benefits and Risks**

It is anticipated that your participation in this project will be meaningful and rewarding to you and will require no longer than approximately one to two hours of your time in the initial interview and any follow-up. In addition, you will be helping my and others’ understanding of the learning strategies and knowledge building of participants with a MRSA diagnosis. It may provide me with the information needed to construct a model for better control and prevention of this condition. Little or no risk to you is anticipated, although it is possible you may find it difficult to discuss personal medical issues. I have 16 years of public health experience and will do my best to help you feel comfortable discussing these issues. This study will be supported financially by a grant I received from the American Society of Clinical Laboratory Science (ASCLS). If you decide to participate in this study, you will receive $100.00 for your time ($50.00 for the first interview and the remaining $50.00 for a follow-up interview, if one is needed).

**Contact Information**

If you have any questions about the research, your rights, and/or research-related injuries to participants, please contact the IRB chair, Dr. Jon Lasser (512-245-3413 – JL@txstate.edu) or the OSP Administrator, Ms. Becky Northcut, at 512-245-2102.

If you have any questions or concerns about this project, please feel free to ask me now.

Thank you for your consideration for participation in this research project. If you agree to participate, please bring this authorization form to the interview session. You will be asked to sign the authorization notice below at that time.

**Authorization:** I have read and understood the description of the above study. I have asked for and received satisfactory explanation of any language that I did not fully understand. I agree to participate in this study, and I understand that I may withdraw my consent at any time. I also understand that the data collected from the interview is intended to be used strictly for analytical, research and educational purposes and I give my permission for release of possible quotes from the interview in the public domain, without my name attached as outlined above. I understand that I will be compensated for my participation as detailed in the above consent form. I have received a copy of this consent form if requested.

_________________________________ _________________

Signature of participant Date

_________________________________

Print name of participant

_________________________________ _________________

Signature of researcher Date

_________________________________

Print name of researcher

## APPENDIX C – INTERVIEW GUIDE

1. Tell me about when you were first diagnosed with MRSA. How did you learn about this and what happened next? [experience and learning]
2. Would you explain how you tried to get a better understanding of MRSA after your diagnosis? What methods did you use to learn more about it? [sources]

*Possible Probes*

*•How information sources influence decision making process*

*•Obstacles and strategies used to overcome them*

*• Role of media as information source*

*• People as sources of information (lay and professional)*

*• Other sources?*

1. What have you learned about MRSA? [knowledge]

*Possible Probes*

•*What does the word infection mean to you?*

*(Risks, how contracted, bodily reaction)*

*•Tell me what you understand about bacterial resistance.*

*(Why it occurs, consequences)*

*•Connection between antibiotic use and spread of infections like MRSA*

*•Control and prevention of resistant infections like MRSA*

1. In what ways do you think your MRSA diagnosis has influenced how you go about making decisions regarding your health? [adaptation and reflection]

*Possible Probes*

*• Usual decision making process?*

*• Differences in decision making style or strategies for health-related life decisions*

1. How have you learned to live with MRSA? [adaptation and reflection]

*Possible Probe*

*•What has helped you come to terms with being MRSA positive?*

1. How has MRSA changed your life? [reflection and closing]
2. What advice would you give to a person you met who had just been diagnosed with MRSA? [experience, learning, sources, adaptation]
3. What advice do you have to offer to the health care arena to help individuals diagnosed with MRSA? [experience, learning, sources, adaptation]

## APPENDIX D – CROSS REFERENCE MATRIX

| **Research Questions** | **Data Collection Sources** | **Subject Category** |
| --- | --- | --- |
| 1. How do participants construct knowledge about MRSA? | - Opening introductions - Interview guide question 1 | - Opening - Experience - Learning |
| 1a. How do participants who have MRSA describe their experiences with learning about MRSA? | - Interview guide question 1 - Interview guide question 7 - Interview guide question 8 | - Experience - Learning |
| 1b. How do participants acquire their knowledge about MRSA? | - Interview guide question 2 - Interview guide question 7 - Interview guide question 8 | - Sources |
| 1c. What understandings do participants have of MRSA and antibiotic resistance? | - Interview guide question 3 - Interview guide question 7 - Interview guide question 8 | - Knowledge |
| 2. How do people adapt to their condition? | - Interview guide question 4 - Interview guide question 7 - Interview guide question 8 | - Adaptation |
| 2a. What strategies for living with MRSA are apparent among those who have a diagnosis of this condition? | - Interview guide question 5 - Interview guide question 7 - Interview guide question 8 | - Adaptation/Reflect |
| 2b. What factors enhance or detract one’s ability about this disease? | - Interview guide question 6 - Interview guide question 7 - Interview guide question 8 - Closing remarks | - Reflect/Closing |
